# Cost-effectiveness of semaglutide 2.4 mg versus liraglutide 3 mg for the treatment of obesity in Greece

**DOI:** 10.3389/fpubh.2025.1690211

**Published:** 2025-10-28

**Authors:** Panagiotis Papantoniou, Nikolaos Maniadakis

**Affiliations:** Department of Public Health Policy, School of Public Health, University of West Attica, Athens, Greece

**Keywords:** cost-effectiveness, obesity, semaglutide, liraglutide, Greece

## Abstract

**Background:**

Obesity is a major public health issue associated with significant humanistic and economic burden. In Greece, liraglutide 3.0 mg is currently the only reimbursed pharmacotherapy for obesity, restricted to patients with morbid obesity and selected comorbidities. Semaglutide 2.4 mg has demonstrated superior clinical efficacy in the STEP-8 clinical trial; however, its cost-effectiveness relative to liraglutide requires further investigation to ensure informed reimbursement decision-making.

**Methods:**

A state-transition model was developed in Microsoft Excel to evaluate the long-term cost-effectiveness of semaglutide 2.4 mg compared with liraglutide 3.0 mg in adults with obesity (BMI ≥ 35 kg/m^2^ and ≥ one weight-related comorbidity). Clinical efficacy and safety inputs were derived from the STEP 8 trial, while cost inputs (expressed in 2025 euros) and utility values were obtained from the literature and published local sources. The analysis was conducted over a 40-year time horizon, with both costs and outcomes discounted at an annual rate of 3.5%. Health outcomes were reported as life-years (LYs) and quality-adjusted life-years (QALYs). The evaluation was conducted from the perspective of the Greek third-party payer, and deterministic, scenario, and probabilistic sensitivity analyses were performed.

**Results:**

Semaglutide 2.4 mg was associated with an incremental mean increase in quality-adjusted life expectancy of 0.09 at modestly incremental higher costs of 1,083 compared with liraglutide 3.0 mg, yielding an incremental cost-effectiveness ratio (ICER) of €12,724 per QALY gained, below the willingness-to-pay threshold of €27,117. Probabilistic sensitivity analysis showed semaglutide dominated liraglutide in 80.8% of simulations (greater QALYs and lower costs) and reached 100% probability of cost-effectiveness at a willingness-to-pay threshold of €9,000 per QALY. Deterministic and scenario analysis identified treatment duration, time horizon, discount rates, and diabetes-related complication costs as key drivers of ICER variability.

**Conclusions:**

Semaglutide 2.4 mg is likely to be a cost-effective treatment option compared to liraglutide 3 mg for patients with a BMI ≥ 35 and at least one weight-related comorbidity in Greece.

## 1 Introduction

Obesity is a major global public health issue, defined as excessive adiposity that adversely affects health and is typically evaluated using body mass index (BMI ≥30 kg/m^2^) (1). Its prevalence has increased sharply since 1990, with an estimated 890 million adults affected worldwide in 2022 and projections indicating that more than 1.2 billion will be living with obesity by 2030 (2). In Greece, 27.98% of adults—approximately 2.5 million individuals—were classified as obese in 2022, placing the country among the highest in Europe (3).

Obesity increases the risk of chronic conditions and is associated with reduced life expectancy, increased all-cause mortality, and diminished health-related quality of life (HRQoL) (4–6). Globally, high BMI accounts for more than 5 million premature deaths and approximately 9% of all disability-adjusted life years (DALYs) annually (7, 8). Beyond its health implications, obesity imposes a considerable economic burden, with its global cost projected to reach USD 2.47 trillion by 2025 (9). In Greece, the total cost of adult obesity was estimated at EUR 4.92 billion in 2024, equivalent to 2.07% of GDP (10).

Lifestyle modification, including diet, physical activity, and behavioral therapy, is the cornerstone of obesity management (11). However, lifestyle interventions alone generally result in modest and challenging-to-maintain weight loss due to physiological mechanisms that favor weight regain (12, 13). Pharmacotherapy, therefore, represents an important adjunct for individuals unable to achieve or maintain sufficient weight reduction. Among currently available agents, glucagon-like peptide-1 (GLP-1) receptor agonists—liraglutide and semaglutide—have shown significant efficacy in reducing weight, improving glycemic control, and lowering cardiometabolic risk factors in the SCALE and STEP clinical programs (14–19).

In Greece, liraglutide 3.0 mg is currently the only reimbursed pharmacotherapy for obesity. Its reimbursement is restricted to adults aged 18–74 years with BMI >40 kg/m^2^ and established CVD or obstructive sleep apnea and requires prior authorization from the National Organization for the Provision of Health Services (EOPYY) (20). Semaglutide 2.4 mg, given its clinical efficacy profile and broad eligible population, is anticipated to generate substantial uptake if reimbursed. Therefore, assessing its cost-effectiveness relative to liraglutide is crucial to inform reimbursement decisions, support HTA evaluations, and optimize the allocation of scarce healthcare resources.

International cost-effectiveness studies consistently show semaglutide 2.4 mg to be cost-effective for chronic weight management. In the UK, a NICE-aligned analysis projected an ICER of £14,827/QALY vs. diet and exercise, with a 90% probability of cost-effectiveness at a £20,000/QALY threshold (21). In Portugal, semaglutide yielded an ICER of €13,459/QALY, with all subgroup ICERs below the conventional threshold of €20,000 (22). In Canada, semaglutide dominated orlistat, naltrexone–bupropion, and liraglutide, with ICERs of CAD 29,014–31,243/QALY vs. standard care from a societal perspective (23). In the US, semaglutide 2.4 mg was cost-effective compared with lifestyle intervention and other branded anti-obesity medications, including liraglutide, with QALY gains of 0.138–0.925 over 30 years and ICERs well below the USD 150,000 threshold (24). Systematic reviews reinforce these findings, whereas more recently, evaluations in patients with obesity and established CVD also found semaglutide cost-effective compared with standard care in the US, Australia, and Canada (25–30).

However, the transferability of existing international findings to the Greek setting remains limited. Most published analyses rely on foreign unit costs and health system structures, while differing in assumptions regarding treatment duration, adherence, discontinuation, and post-treatment weight regain. In Greece, economic evidence is currently limited to a short-term cost-effectiveness analysis, which demonstrates favorable results for semaglutide vs. liraglutide over a 68-week horizon (31), without capturing long-term outcomes. Similarly, a recent evaluation from Egypt confirmed the short-term cost-effectiveness of semaglutide compared with liraglutide, further reinforcing its economic value across diverse healthcare contexts (32).

The objective of this study is therefore to evaluate the long-term cost-effectiveness of semaglutide 2.4 mg compared with liraglutide 3.0 mg for adults with obesity (BMI ≥ 35 kg/m^2^ and ≥ one weight-related comorbidity) in Greece, from the perspective of the third-party payer (EOPYY).

## 2 Materials and methods

### 2.1 Model structure and description

A Markov state-transition model was developed in Microsoft Excel (Microsoft Corp, Redmond, WA, USA) to estimate the long-term cost-effectiveness of semaglutide 2.4 mg compared with liraglutide 3.0 mg for the treatment of adults in Greece with a BMI greater than 35 kg/m^2^ and at least one weight-related comorbidity. Liraglutide 3.0 mg was selected as the comparator because it is the only reimbursed obesity pharmacotherapy in Greece, while other anti-obesity agents, such as naltrexone–bupropion and tirzepatide, were excluded as they are paid for exclusively out-of-pocket.

Unlike more complex multi-biomarker frameworks such as the Core Obesity Model (COM) (21–24), the present model adopted a parsimonious structure in which the BMI served as the sole surrogate risk factor. Changes in BMI under treatment or natural progression were dynamically translated into the incidence of obesity-related complications using published risk equations or transition probabilities. This simplified approach was chosen to maximize transparency, reproducibility, and adaptability to Greek decision-making while still capturing the principal health and economic consequences of obesity. It is critical to note that while multi-biomarker models, such as the COM, simultaneously capture changes in additional surrogate endpoints, including blood pressure, lipids, and glycemic control, this analysis employed BMI as the sole surrogate risk factor for three reasons. First, it is a well-established and consistently the strongest and most validated predictor of obesity-related outcomes, including type 2 diabetes, cardiovascular disease, and mortality (33, 34). Second, BMI is systematically collected in both clinical trials and population-based epidemiological studies, and is the only measure for which robust, representative, and longitudinal data are consistently available in the Greek setting (35, 36). This availability allows the model to be parameterised and externally validated with local data, thereby enhancing its credibility and relevance for Greek decision-makers. Third, several previously published cost-effectiveness studies of obesity pharmacotherapies have successfully applied BMI-only frameworks, producing reliable results that are broadly consistent with multi-biomarker models and policy-relevant in high-income settings (37–40). These precedents demonstrate that a BMI-driven structure is a methodologically accepted and pragmatic approach for long-term decision modeling. Nonetheless, as discussed later, future research should validate the findings of the present single-biomarker model against multi-biomarker frameworks to confirm robustness under alternative structural assumptions.

The model followed a hypothetical cohort of 1,000 adults whose baseline characteristics were aligned with those of the STEP 8 clinical trial population (41), supplemented with clinical expert opinion where local adjustment was necessary. Health states included no complication, single complications (acute coronary syndrome, type 2 diabetes, hypertension, dyslipidemia, asthma, chronic kidney disease, and sleep apnea), combined complications, and death. All patients enter the model in the “no complications” state, characterized by their baseline BMI, demographic characteristics, and utility value. During each cycle, individuals could transition from the non-complication state to one of the single-complication states, representing the first onset of type 2 diabetes, acute coronary syndrome (myocardial infarction or unstable angina), hypertension, dyslipidemia, asthma, sleep apnea, or chronic kidney disease. From there, patients could progress into multi-morbidity states, defined as the coexistence of two or three of these comorbidities, thereby reflecting the clustering of obesity-related diseases observed in real-world populations. All health states were linked to an absorbing death state, allowing transitions from any condition to mortality according to age-, sex-, and risk-adjusted probabilities.

The model was run over a 40-year time horizon to approximate a lifetime perspective, with 3-month cycles in the first year (to capture treatment response and discontinuation) and annual cycles thereafter. A half-cycle correction was applied to reflect the mid-cycle timing of events. Both interventions were assumed to be administered for a maximum of 2 years, with a non-responder stopping rule at 12 weeks (failure to achieve ≥5% weight loss from baseline) applied in line with STEP 8 (41). Following discontinuation, patients reverted to the diet-and-exercise trajectory, and treatment benefits diminished progressively until values returned to baseline (21–24).

**Figure 1:**
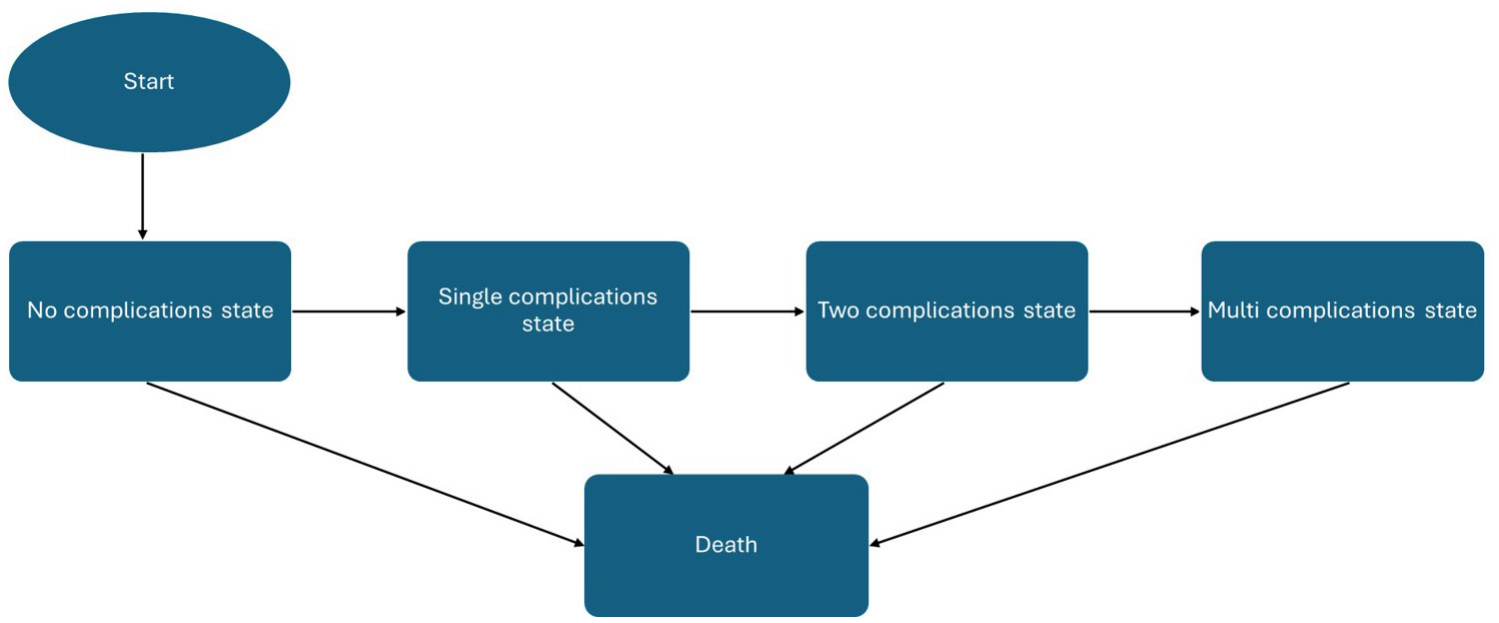
Graphic depiction of the economic model.

Outcomes were expressed in terms of costs, life-years (LYs), quality-adjusted life-years (QALYs), and incremental cost-effectiveness ratios (ICERs). Costs and outcomes were discounted at an annual rate of 3.5%, consistent with prior published Greek cost-effectiveness studies (42–46), as no official national HTA guideline currently specifies a discounting rate. Cost-effectiveness was assessed against a willingness-to-pay (WTP) threshold of €27,117 per QALY gained, with an additional threshold of €34,000 per QALY gained also examined, reflecting the only two published estimates available for Greece (47, 48). A schematic of the model structure is presented in Figure 1. The analysis was conducted in line with the Consolidated Health Economic Evaluation Reporting Standards (CHEERS) 2022 guidelines (49).

### 2.2 Cohort of patients

The modeled population reflects individuals eligible for obesity pharmacotherapy under the criteria of the Greek National Organization for the Provision of Health Services (EOPYY) (20), specifically adults with a BMI greater than 40 kg/m^2^ and at least one weight-related comorbidity. Baseline characteristics were sourced from the STEP-8 trial, the only randomized controlled trial directly comparing semaglutide 2.4 mg with liraglutide 3.0 mg in obesity, thereby providing robust and internally consistent data for this head-to-head evaluation. Recognizing that the STEP-8 population may not fully represent the Greek obesity population, trial-based characteristics were reviewed and adapted using local epidemiological data and expert clinical opinion (e.g., smoking prevalence, hypertension, and diabetes distribution) to enhance relevance for the Greek setting. This approach ensured internal validity while improving external applicability, though it is acknowledged that comprehensive local baseline data remain limited.

The simulated cohort had a mean age of 48.6 years, a mean BMI of 41.5 kg/m^2^, and was predominantly female (73.6%). Cardiometabolic risk factors included a mean systolic blood pressure of 128.4 mmHg, a mean total cholesterol level of 186.0 mg/dL, a mean HDL-cholesterol level of 50.9 mg/dL, and a mean triglyceride level of 128.2 mg/dL. Smoking prevalence was assumed at 45.2%, while 31.4% and 44.7% of patients were on lipid-lowering and anti-hypertensive therapy, respectively. A complete list of baseline cohort characteristics is provided in Table 1.

**Table 1:** Baseline patients' characteristics.

**Parameters**	**Mean**	**Source**
Age (years)	48.6	STEP-8 trial (31)
BMI (kg/m^2^)	41.5	STEP-8 trial (31)
Height (m)	1.70	STEP-8 trial (31)
SBP (mmHg)	128.40	STEP-8 trial (31)
T-chol (mg/dL)	186.00	STEP-8 trial (31)
HDL-chol (mg/dL)	50.90	STEP-8 trial (31)
HbA1c from onset of T2D (%-points)	7.50%	Clinical expert opinion
T2D duration (years)	6.00	Clinical expert opinion
Triglycerides (mg/dL)	128.20	STEP-8 trial (31)
Proportion triglyceride ≥150 mg/dL (%)	28.10	STEP-8 trial (31)
Smokers (%)	45.20	Clinical expert opinion
Females (%)	73.60	STEP-8 trial (31)
On lipid-lowering medication (%)	31.4	STEP-8 trial (31)
On anti-hypertensive medication (%)	44.7	Clinical expert opinion
**Glycaemic status at baseline**		
Proportion with no pre-T2DM, T2DM	40.40	Clinical expert opinion
Proportion with pre-T2DM	48.80	STEP-8 trial (31)
Proportion with T2DM	10.80	Clinical expert opinion
History of CVD at baseline (%)	5.0	Clinical expert opinion

HbA1c, Hemoglobin A1c; T-chol, Total cholesterol; HDL-chol, High density lipoprotein cholesterol; SBP, Systolic blood pressure; T2D, Type 2 diabetes mellitus; coronary artery disorders, including coronary artery disease, angina pectoris, myocardial infarction, acute myocardial infarction, myocardial ischemia, arteriosclerosis of the coronary artery, acute coronary syndrome, angina unstable, coronary artery stenosis, microvascular coronary artery disease, arteriospasm of the coronary; cardiovascular disease.

### 2.3 Clinical efficacy and safety

Treatment efficacy for semaglutide 2.4 mg and liraglutide 3.0 mg was represented by their effect on BMI, with clinical inputs derived from the STEP-8 trial (full analysis set) (41) (Supplementary Table 1). A maximum treatment duration of 2 years was assumed, with efficacy estimates based on an intention-to-treat analysis using the treatment-policy estimand, thereby capturing population-level effects irrespective of adherence or treatment modifications (41). A non-responder stopping rule was applied at 12 weeks, defined as failure to achieve ≥5% weight loss from baseline. Non-responder rates were 24.6% for semaglutide and 38.6% for liraglutide. These patients were assumed to continue with diet and exercise alone, with efficacy values informed by the corresponding arm of STEP-8 (41).

Following treatment discontinuation (after 2 years, or earlier due to non-response or adverse events), treatment effects were assumed to decline according to a catch-up rate until BMI returned to baseline. Thereafter, BMI was assumed to increase at a natural rate of 0.47 kg/year, based on longitudinal data from the UK General Practice Research Database reported by Ara et al. (50). This approach has been widely adopted in prior obesity modeling studies and in economic evaluations of semaglutide and other pharmacotherapies (21–24). To address uncertainty around post-treatment weight trajectories, additional scenarios were tested, including accelerated weight regain to baseline within 1 year and immediate reversion to natural progression without residual diet-and-exercise benefit.

Safety inputs were also derived from STEP-8 (41). Treatment-related adverse events (AEs) captured in the model included gastrointestinal events (e.g., nausea, vomiting, diarrhea) and non-severe hypoglycaemia. These were applied during active treatment, with discontinuations due to AEs transitioning patients onto the diet-and- exercise pathway.

### 2.4 Transition probabilities

Consistent with previous obesity modeling studies (21–24), transition probabilities between health states and the incidence of obesity-related complications were derived from published risk equations that link changes in BMI to the risk of developing comorbidities. Specifically, the incidence of T2D was estimated using the QDiabetes risk prediction algorithm, a validated tool based on large UK primary care cohorts (51). The risk of ACS was estimated using the QRisk3 equation, which integrates demographic and clinical risk factors (52). For individuals with T2D, the probability of recurrent coronary events was derived from the Framingham Recurrent CHD equations (53). The incidence of dyslipidemia was estimated from the PERSINA Guilan Cohort study (54), while the risk of hypertension was predicted using longitudinal data from the Johns Hopkins Precursors Study (55). The probability of asthma onset was informed by analyses from the National Health and Nutrition Examination Survey (56). The incidence of sleep apnea was estimated using pooled estimates from a systematic review and meta-analysis (57). Finally, the risk of chronic kidney disease (CKD) was informed by prospective analyses of a primary care cohort of 1.4 million adults in England (58).

### 2.5 Mortality

Obesity is associated with increased risk of premature mortality, particularly through complications such as T2D and CVD (59). Age- and sex-specific all-cause mortality rates for the general population were obtained from the WHO life tables for Greece (60). We followed the approach adopted by Miguel et al. (22) to avoid double counting of mortality attributable to obesity-related complications. Consequently, all-cause mortality was adjusted by subtracting deaths due to specific modeled diseases. The resulting disease-specific mortality was then modified using hazard ratios for BMI, derived from an extensive cohort study based on the UK Clinical Practice Research Datalink (CPRD) (61). These HRs capture the residual mortality risk associated with higher BMI levels that are not otherwise explained by the explicitly modeled comorbidities (22). Full parameter values for BMI-related hazard ratios and mortality adjustments are reported in Supplementary Table 2.

### 2.6 Utilities

Baseline HRQoL was estimated using a regression-based function of BMI, derived from baseline data in the STEP 8 trial, an approach also adopted in previous cost-effectiveness studies (21–24). Utility scores were regressed against baseline BMI, controlling for age, sex, coronary artery disease, prediabetes, hypertension, and smoking status. This generated a baseline, complication-free utility value that varied with BMI across cycles, consistent with prior obesity economic models (21–24). The explicit regression equation applied in the model was: “*Baseline Utility* = *0.942975 – 0.0005414* ^*^Age – 0.0742818 ^*^HeartCirc – 0.0097531 ^*^Hypertension + *0.0039044* ^*^Smoke_Current – 0.0081972 ^*^ *Smoke_Previous* + *0.0065954* ^*^BMI – 0.0002476 ^*^ *BMI*^2^ + *0.00000175* ^*^ *BMI*^3^ –* 0.0031133* ^*^ *Prediabetes*” (Supplementary Table 3).

Utilities were dynamically updated in each cycle to reflect both BMI trajectories and the onset of comorbidities. As individuals transitioned into health states corresponding to obesity-related complications, condition-specific disutilities were applied additively to the baseline utility. Disutilities for chronic comorbidities were obtained from published literature (62–66). For acute events such as stroke or transient ischemic attack, temporary disutilities were applied in the cycle of occurrence. Treatment-related adverse events, primarily gastrointestinal symptoms and non-severe hypoglycaemia, were also incorporated as short-duration disutilities during active therapy, consistent with prior studies (21–24). The baseline utilities and disutilities applied in the model are reported in Table 2.

**Table 2:** Baseline utility and disutilities in health states and acute events.

**Parameters**	**Mean**	**SE**
**Utility at baseline**		
Baseline utility	0.78	0.023
**Disutility applied in the health state**		
T2D	−0.029	0.006
ACS	−0.037	0.008
OSA	−0.013	0.004
Asthma	−0.021	0.005
Dyslipidaemia	−0.037	0.004
Hypertension	−0.014	0.003
Chronic kidney disease	−0.049	0.002
**Disutility per event**		
ACS	−0.129	0.032
Stroke	−0.181	0.045
Transient Ischemic Attacks	−0.033	0.008
Severe GI Events	−0.001	0.0002
Severe Hypoglycaemia	−0.015	0.002
Non-severe Hypoglycaemia	−0.0062	0.004

T2D, type-2 diabetes; ACS, acute coronary syndrome; OSA, obstructive sleep apnea; GI, gastrointestinal.

### 2.7 Costs

The analysis was conducted from the perspective of the Greek third-party payer (EOPYY) and therefore included only direct medical costs. Indirect costs, such as productivity losses, were excluded from the analysis. Confidential, mandatory or voluntary discounts were not applied as such data are confidential and not readily accessible.

Drug acquisition costs for semaglutide 2.4 mg and liraglutide 3.0 mg were obtained from the most recent official price bulletin (67). Payer costs were derived by applying the statutory 25% co-payment to retail prices, consistent with EOPYY reimbursement rules. The annual cost of obesity monitoring comprised three general practitioner visits, four visits to an obesity or surgical specialist, two visits to a dietitian/nutritionist, and three complete blood and urine panels per patient per year. Unit costs for visits and diagnostic tests were extracted from the Government Gazette and the EOPYY reimbursement schedule (68, 69). Costs associated with the treatment of obesity-related complications and with adverse events were sourced from published Greek literature and national unit cost databases. Acute event costs—including hospitalizations for acute coronary syndrome, stroke, or hypoglycemia—were retrieved from publicly available Diagnosis-Related Groups (DRGs) (70). All costs were expressed in 2025 euros. Where necessary, earlier cost estimates were adjusted to 2025 values using the Consumer Price Index (CPI) published by the Hellenic Statistical Authority (ELSTAT) (71). Table 3 illustrates the cost inputs used in the present analysis.

**Table 3:** Health state, event, drug acquisition and consumable costs used in the analysis, expressed in 2025 Euros (€).

**Cost description**	**Costs (€)**	**Reference**
**Costs of interventions**		
Semaglutide 0.25 mg	123.62	Drug price bulletin (67)
Semaglutide 0.5 mg	123.62	
Semaglutide 1 mg	123.62	
Semaglutide 1.7 mg	167.93	
Semaglutide 2.4 mg	190.21	
Liraglutide 3 mg	162.98	
**Costs of consumables (annual)**		
Needles for injectable drugs	33.58	Government Gazette (FEK B' 4045/17-11-2017) (73)
**Monitoring costs (annual)**		
Monitoring obesity costs	124.88	Experts' opinion and Government Gazette (67–69)
**Adverse event costs (acute)**		
Minor hypoglycemia	287.77	Tzanetakos et al. (74)
Major hypoglycemia	805.77	Tzanetakos et al. (74)
Major gastrointestinal event	654.50	Government Gazette (DRGs-code: Π41X and Π41X) (70)
**Health state costs (annual)**		
Non complications	124.88	Experts' opinion & Government Gazette (69, 70)
Type-2 diabetes	1,125.91	Athanasakis et al. (75)
Hypertension	593.84	Tsalta et al. (76)
Chronic kidney disease	811.46	Stafylas et al. (77)
Sleep apnea	1,688.40	Prapa et al. (78)
Dyslipidemia	924.21	Migdalis et al. (79)
Asthma	796.58	Vellopoulou et al. (80)
Acute coronary syndrome	1,819.31	Tzanetakos et al. (74)
**Acute care costs**		
Myocardial infarction (fatal)	4,166.00	Government Gazette (DRGs-code: K10M) (70)
Myocardial infarction (non-fatal)	2,724.00	Government Gazette (DRGs-code: K10X) (70)
Angina (fatal)	940.00	Government Gazette (DRGs-code: K47M) (70)
Angina (non-fatal)	424.00	Government Gazette (DRGs-code: K47X) (70)
Stroke (fatal)	2,475.00	Government Gazette (DRGs-code: N30MA) (70)
Stroke (non-fatal)	1,625.00	Government Gazette (DRGs-code: N30MB) (70)
Transient ischemic event	806.50	Government Gazette (DRGs-codes: N29M & N29X) (70)

### 2.8 Sensitivity analyses

Deterministic sensitivity analyses (DSA) were conducted to assess the impact of parameter uncertainty on model outcomes by varying one parameter at a time while holding all others constant. Key clinical and economic inputs—including drug acquisition costs, complication costs, utility values, discount rate, and assumptions regarding post-treatment weight regain—were varied. When empirical measures of parameter uncertainty (e.g., standard errors or confidence intervals) were not available from published sources, a conservative range of ±25% was applied around the base-case value. This approach follows established practice in cost-effectiveness modeling and aligns with previous economic evaluations in obesity (21–24). The eleven most influential parameters were identified and ranked according to their effect on the incremental cost-effectiveness ratio (ICER), with results presented in a tornado diagram.

A series of scenario analyses was conducted to explore the impact of alternative input parameters and structural assumptions on cost-effectiveness outcomes. These included: restricting to disease-specific mortality only, extending treatment duration beyond the base case to 3, 4, 5, 6, and 9 years; applying alternative efficacy estimands, namely the trial product estimand (with and without treatment discontinuation for semaglutide 2.4 mg); and modifying post-treatment trajectories, with scenarios assuming reversion to natural progression without the benefit of diet and exercise, as well as accelerated catch-up with treatment effects fading completely within 1 year.

Probabilistic sensitivity analysis (PSA) was undertaken to explore joint parameter uncertainty and estimate the probability that semaglutide 2.4 mg is cost-effective compared with liraglutide 3.0 mg. A Monte Carlo simulation with 1,000 iterations was implemented, drawing inputs from predefined probability distributions consistent with recommended practice, namely Gamma for costs, Beta for probabilities and utilities, and Normal or Lognormal for relative risks and treatment effects (72). Standard errors were taken from original data sources when reported; in their absence, a standard error of 20% of the point estimate was assumed.

PSA outcomes were summarized on a cost-effectiveness plane and through a cost-effectiveness acceptability curve (CEAC). Results were reported as mean costs, QALYs, and ICERs across simulations, with 95% confidence intervals derived using the percentile method.

## 3 Results

### 3.1 Base case analysis

Total discounted lifetime costs, life-years (LYs), quality-adjusted life-years (QALYs), and incremental cost-effectiveness ratios (ICERs) are presented in Table 4. Over a 40-year horizon, semaglutide 2.4 mg was associated with higher total direct medical costs than liraglutide 3.0 mg (€27,731 vs. €26,647; incremental €1,083). This difference was primarily attributable to higher drug acquisition costs in the semaglutide arm (€10,261 vs. €8,844; +€1,417). Monitoring costs were nearly identical between groups (€2,172 vs. €2,166). Significantly, these additional treatment costs were partially offset by reductions in downstream complications. Compared with liraglutide, semaglutide reduced the costs of obesity-related disease states (€14,828 vs. €15,165; –€337) and event-related complications (€469 vs. €472; –€3).

**Table 4:** Base case cost-effectiveness results for semaglutide 2.4 mg vs. liraglutide 3 mg.

**Parameters**	**Semaglutide 2.4 mg**	**Liraglutide 3 mg**	**Incremental**
Discounted total lifetime direct medical costs	27,730.63	26,647.26	1,083.37
Obesity treatment costs	10,261.39	8,843.97	1,417.42
Obesity monitoring costs	2,172.22	2,166.16	6.05
Obesity complications: state costs	14,827.84	15,165.16	−337.31
Obesity complications: event costs	469.18	471.97	−2.79
Discounted quality-adjusted life expectancy (QALYs)	13.90	13.81	0.09
Discounted life expectancy (LYs)	17.06	17.03	0.04
Incremental cost-effectiveness ratio (€per QALY gained)			12,724
Incremental cost-effectiveness ratio (€per LY gained)			28,051
NMB (QALYs) WTP: €27,117 per QALY			1,357
NMB (QALYs) WTP: €30,000 per QALY			1,617

QALY, quality-adjusted life-year; LY, life-year; ICER, incremental cost-effectiveness ratio; NMB, net monetary benefit.

Health outcomes favored semaglutide, which provided an additional 0.09 QALYs (13.90 vs. 13.81) and 0.04 LYs (17.06 vs. 17.03). The resulting ICERs were €12,724 per QALY gained and €28,051 per LY gained. At the WTP threshold of €27,117 per QALY, semaglutide yielded a positive net monetary benefit (NMB) of €1,357. When applying a threshold of €34,000 per QALY, as proposed by recent research for non-oncology interventions in Greece (47), the NMB increased to €1,616.

### 3.2 Scenario analysis

Scenario analyses (Table 5) confirmed the robustness of the base case findings. Extending treatment duration from 3 to 9 years resulted in ICERs ranging from €14,020 to €15,487 per QALY. The application of the trial product estimand with a 12-week stopping rule resulted in a slight improvement in cost-effectiveness (ICER: €12,331/QALY), whereas removing the non-responder stopping rule increased the ICER to €19,249/QALY. Post-treatment assumptions had a limited impact on the cost-effectiveness results, with reversion to no-treatment progression resulting in an ICER of €12,669/QALY, while accelerated weight regain to baseline within 1 year yielded an ICER of €12,410/QALY. Finally, restricting the model to disease-specific mortality (excluding BMI-dependent excess mortality) increased the ICER to €16,071/QALY. Overall, semaglutide remained cost-effective vs. liraglutide across all structural and clinical assumptions, reinforcing the robustness of the base case findings.

**Table 5:** Scenario analysis results for semaglutide 2.4 mg vs. liraglutide 3 mg.

**Scenarios**		**Total costs**		**Total QALYs**		**ICER**	**% Change vs. base case**
		**Semaglutide 2.4 mg**	**Liraglutide 3 mg**	**Semaglutide 2.4 mg**	**Liraglutide 3 mg**	**Semaglutide 2.4 mg versus Liraglutide 3 mg**	
Base case		27,730	26,647	13.90	13.81	12,724	
1.	Disease-specific mortality only	27,461	26,497.	15.12	15.06	16,071	+26%
2.	Treatment duration: 3 years	28,529	26,847	13.97	13.85	14,020	+10%
3.	Treatment duration: 4 years	29,392	27,047	14.05	13.89	14,655	+15%
4.	Treatment duration: 5 years	30,250	27,247	14.12	13.92	15,018	+18%
5.	Treatment duration: 6 years	31,104	27,447	14.20	13.96	15,239	+20%
6.	Treatment duration: 9 years	31,983	27,647	14.29	14.01	15,487	+22%
7.	Trial product estimand with a stopping rule	27,733	26,747	13.89	13.81	12,331	−3%
8.	Trial product estimand without a stopping rule	28,822	26,897	13.91	13.81	19,249	+51%
9.	Post-treatment discontinuation to no treatment	27,584	26,697	13.89	13.82	12,669	−0.4%
10.	Weight returns to baseline in 1 year	27,590	26,722	13.83	13.76	12,410	−2%

### 3.3 Sensitivity analysis

**Figure 2:**
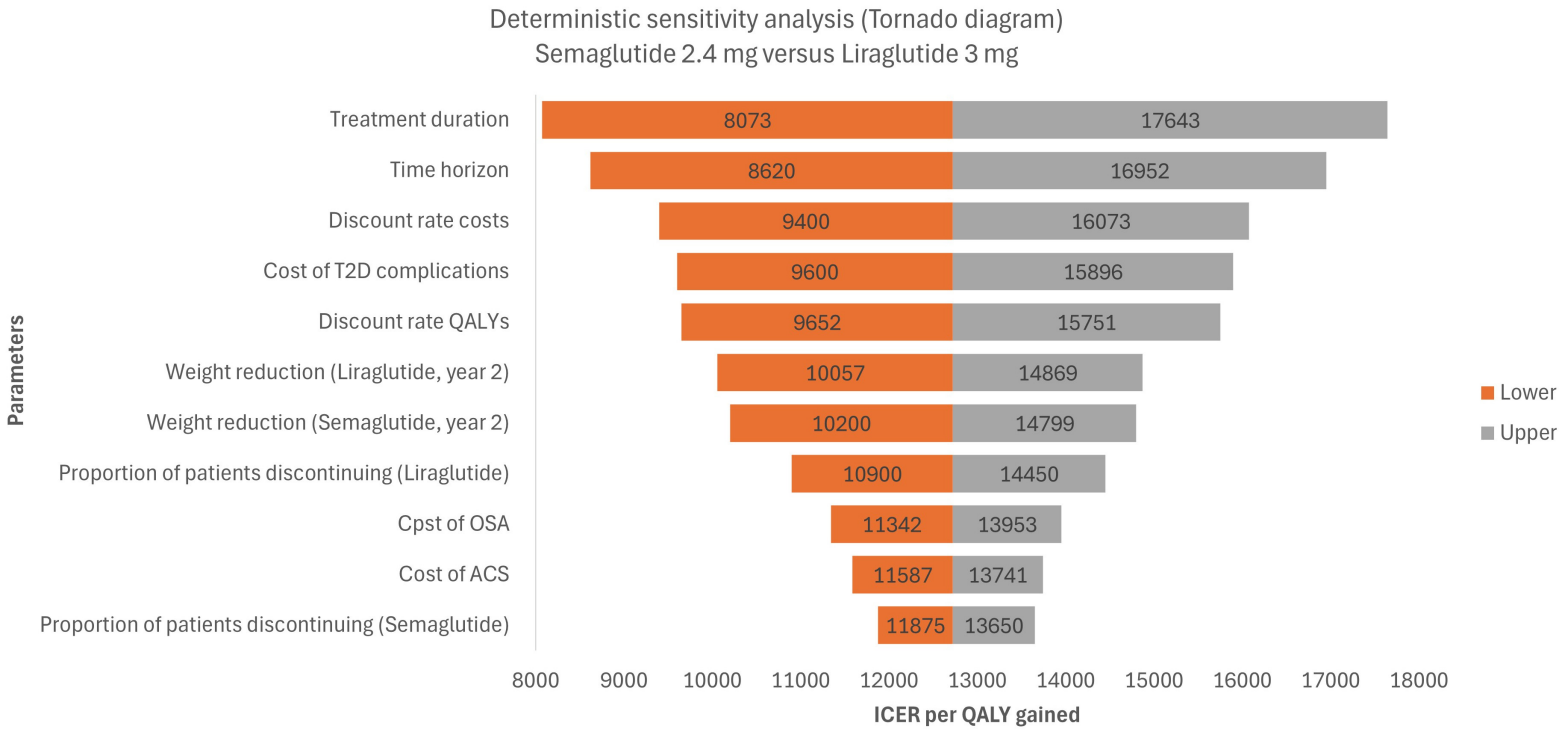
Tornado diagram based on a deterministic-sensitivity analysis for semaglutide 2.4 mg vs. liraglutide 3 mg. T2D, type-2 diabetes; QALY, quality-adjusted life year; OSA, obstructive sleep apnea; ACS, acute coronary syndrome; ICER, incremental cost-effectiveness ratio.

The results of the DSA are presented in Figure 2 in the form of a tornado graph illustrating the 11 most influential parameters impacting the base-case ICER. It is worth noting that in all DSA scenarios, the resulting ICER remained below the WTP threshold of €27,117/QALY.

The parameters with the most significant influence on cost-effectiveness were treatment duration, time horizon, discount rates, and the cost of T2D complications. Extending treatment duration or shortening the time horizon increased the ICER (up to €17,643). Conversely, reducing treatment duration or extending the time horizon improved cost-effectiveness (ICERs as low as €8,073–€8,620). Discounting assumptions were also important: varying the discount rates for costs and QALYs shifted the ICER between €9,400 and €16,073 for costs and €9,652 to €15,751 for QALYs. Similarly, varying the cost of T2D complications yielded ICERs ranging from €9,600 to €15,896.

The results of the probabilistic sensitivity analysis (PSA) are summarized in Table 6. Across 1,000 Monte Carlo simulations, semaglutide 2.4 mg was associated with lower mean lifetime costs (€24,902 vs. €25,203 for liraglutide 3.0 mg) and higher mean QALYs (14.77 vs. 14.70). This translated into mean incremental cost savings of €301 and incremental health gains of 0.07 QALYs in favor of semaglutide. A decomposition of incremental costs is presented in Supplementary Table 4. In the deterministic base case, semaglutide incurred higher total lifetime costs (€1,083) compared with liraglutide, primarily due to higher drug acquisition expenses (€1,417), which were only partially offset by lower costs associated with obesity complications (€340 in total). In contrast, the probabilistic sensitivity analysis (PSA) produced a mean incremental cost saving (–€301). This difference reflects the incorporation of parameter uncertainty in the PSA, where joint sampling across treatment effects, complication risks, and cost inputs allowed scenarios in which semaglutide achieved larger reductions in obesity-related complications. On average, savings in complication-related “state” costs (–€1,287.99) and event costs (–€14.14) outweighed the higher drug and monitoring costs (+€997.23 and +€4.25, respectively). This resulted in a net mean saving of €300.65 for semaglutide, demonstrating that the cost-effectiveness of semaglutide remains robust when uncertainty and inter-parameter variability are considered.

**Table 6:** Probabilistic sensitivity analysis results for semaglutide 2.4 mg vs. liraglutide 3 mg.

**Parameters**	**Semaglutide 2.4 mg**		**Liraglutide 3 mg**		**Incremental**	
	**Costs (**€**)**	**QALYs**	**Costs (**€**)**	**QALYs**	**Costs (**€**)**	**QALYs**
B-Mean	24,902.28	14.77	25,202.93	14.70	−300.65	0.07
B-SD	1,479.58	0.18	1,498.27	0.17	330.05	0.01
B-95% LCI	21,987.93	14.43	22,346.25	14.37	−942.68	0.04
B-95% UCI	27,957.94	15.11	28,335.21	15.04	334.89	0.09
B-Min	20,104.68	14.21	20,773.60	14.14	−1,215.77	0.03
B-Max	30,381.04	15.30	30,474.66	15.22	759.03	0.10

B, Bootstrap; SD, standard deviation; LCI, lower confidence interval; UCI, upper confidence interval.

Results were based on 1,000 non-parametric bootstrap experiments.

Uncertainty around these estimates was modest. The standard deviation of incremental costs was €330, with a 95% confidence interval (CI) ranging from €−943 to €335. For incremental QALYs, the standard deviation was 0.01, with a 95% CI of +0.04 to +0.09. Across all simulations, semaglutide consistently generated positive incremental QALYs (minimum +0.03, maximum +0.10).

**Figure 3:**
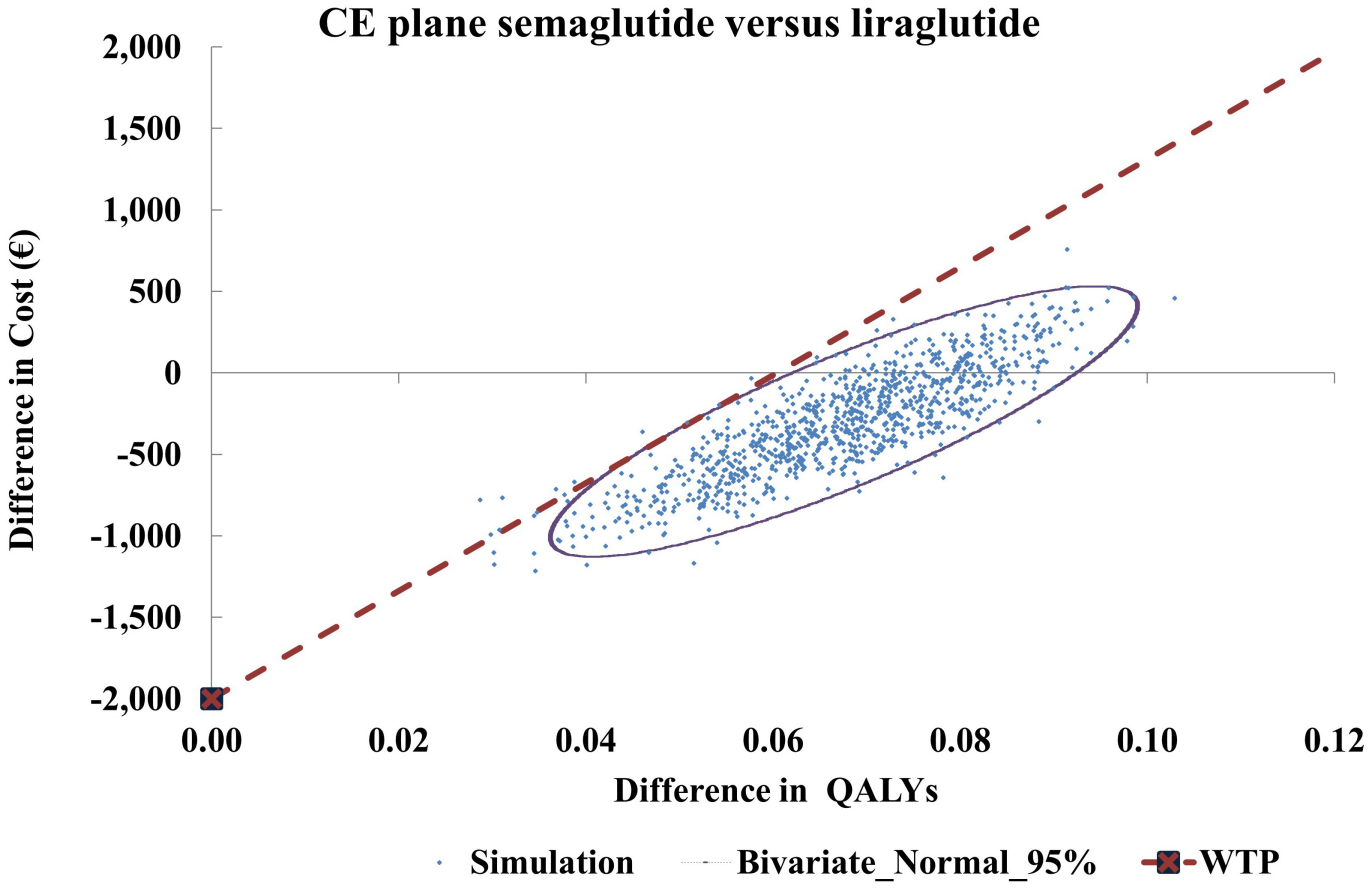
Cost-effectiveness plane for semaglutide 2.4 mg vs. liraglutide 3 mg. CE, cost-effectiveness; ICER, incremental cost-effectiveness ratio; QALY, quality-adjusted life-year; WTP, willingness-to-pay.

The cost-effectiveness (CE) plane (Figure 3) illustrates that 80.8% of simulations fell in the south-east quadrant, where semaglutide dominated liraglutide (more QALYs at lower costs). The remaining simulations were in the north-east quadrant, where semaglutide provided more QALYs but at higher costs. No simulations indicated fewer QALYs with semaglutide.

**Figure 4:**
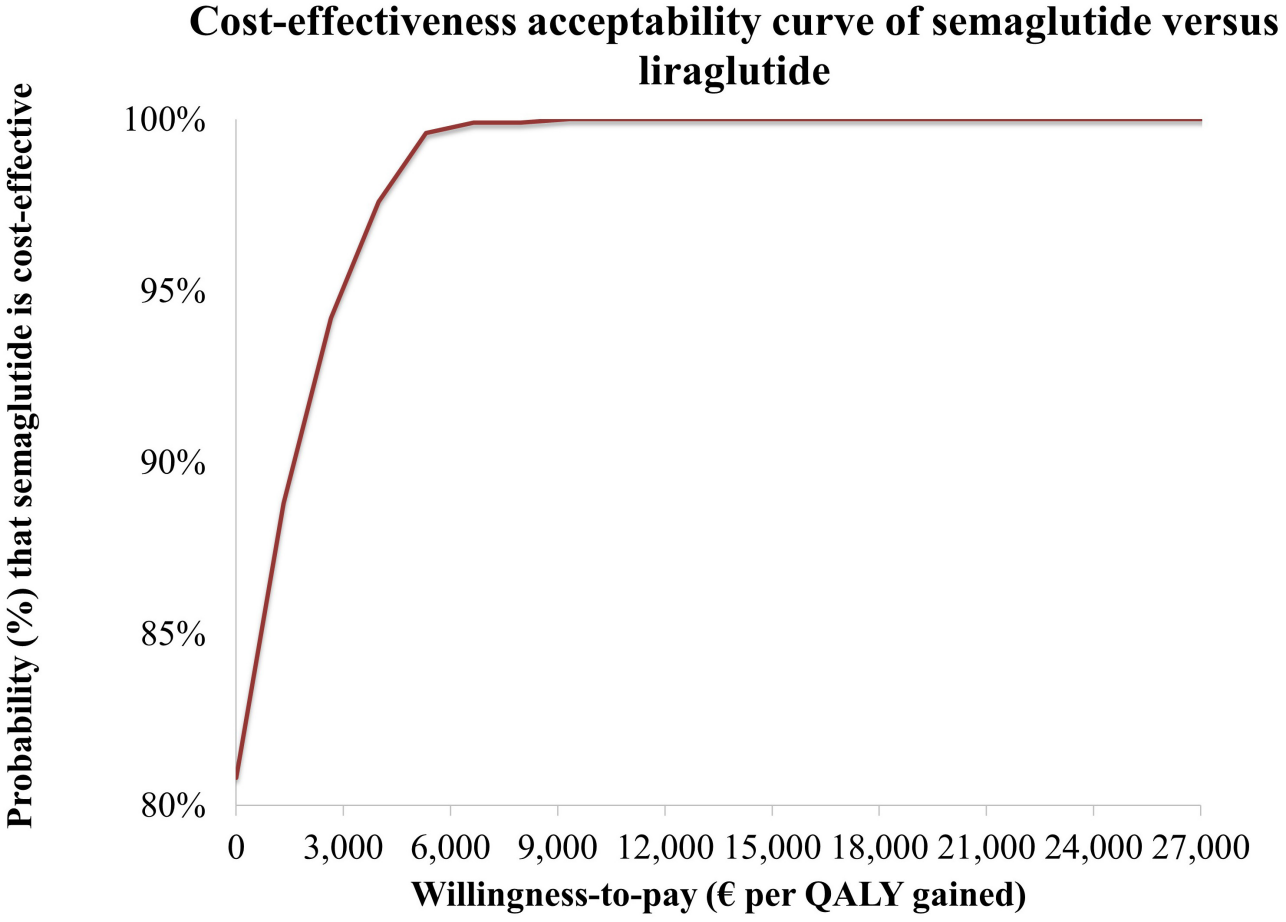
Cost-effectiveness acceptability curve (CEAC) for semaglutide 2.4 mg vs. liraglutide 3 mg.

Consistent with these results, the cost-effectiveness acceptability curve (CEAC) (Figure 4) showed that semaglutide achieved a positive net monetary benefit (NMB) in 80.8% of simulations. The probability of semaglutide being cost-effective reached 100% at a WTP threshold of approximately €9,000 per QALY gained. At the examined Greek thresholds of €27,117 or €34,000 per QALY, semaglutide was cost-effective vs. liraglutide in all simulations.

## 4 Discussion

This study presents the first long-term cost-effectiveness analysis of semaglutide 2.4 mg compared with liraglutide 3.0 mg for the treatment of adults with obesity (BMI ≥ 35 kg/m^2^ and at least one comorbidity) from the perspective of the Greek third-party payer. Using a state-transition model over a 40-year horizon, semaglutide was shown to be cost-effective relative to liraglutide, with an ICER of €12,724 per QALY gained, well below the commonly used Greek WTP thresholds. The probabilistic sensitivity analysis confirmed the robustness of these findings, with semaglutide dominating liraglutide in most simulations, generating greater QALYs and lower costs in 80.8% of cases, and achieved a 100% probability of cost-effectiveness at a WTP threshold of approximately €9,000 per QALY. The findings remained robust when evaluated against various cost-effectiveness thresholds (€27,117 and €34,000 per QALY which reinforced semaglutide's cost-effectiveness profile and strengthened the policy relevance of the analysis for HTA and reimbursement decision-making. Deterministic and scenario analyses further demonstrated that the results were most sensitive to assumptions regarding treatment duration, time horizon, discounting, and the costs of diabetes-related complications; however, semaglutide remained cost-effective in all scenarios.

Our results align with a growing international evidence base. Cost-effectiveness studies conducted in the UK, Portugal, Canada, and the United States consistently concluded that semaglutide is cost-effective relative to lifestyle management and other pharmacotherapies, including liraglutide (21–27). In Portugal, semaglutide yielded an ICER of €13,459 per QALY compared with diet and exercise alone in adults with BMI >30 and comorbidities, with a 100% probability of cost-effectiveness at a €20,000 threshold (22). In the UK, Sandhu et al. (21) reported that semaglutide was cost-effective in 90% of simulations at a willingness-to-pay threshold of £20,000 per QALY, whereas Olivieri et al. (23) demonstrated semaglutide to be cost-effective vs. liraglutide and other agents in Canada, with extended dominance over naltrexone–bupropion. Our results are consistent with the findings of Alshahawey et al. (32), who conducted a decision analysis in the Egyptian setting and reported semaglutide 2.4 mg as a cost-effective and clinically superior alternative to liraglutide 3.0 mg for achieving significant weight loss. It is noteworthy that the probabilistic analysis yielded mean cost savings despite the deterministic base case showing marginally higher costs for semaglutide. This discrepancy reflects the probabilistic sampling of interdependent parameters—treatment effects, complication risks, and healthcare costs—enabling scenarios where greater reductions in obesity-related complications result in substantial downstream savings. The resulting net cost savings observed in the PSA further reinforce the robustness of semaglutide's cost-effectiveness under uncertainty.

The present analysis builds on this literature by providing country-specific evidence for Greece, where liraglutide remains the only reimbursed obesity pharmacotherapy and is restricted to adults with morbid obesity and established cardiovascular disease or sleep apnoea. Methodologically, this study advances previous work by broadening the scope of obesity-related complications considered. While earlier models often focused on type 2 diabetes, cancer and cardiovascular disease (20–23), we also incorporated chronic kidney disease, sleep apnoea, hypertension, asthma, and dyslipidaemia, thereby capturing a broader spectrum of obesity-related morbidity. This increases face validity and decision-making relevance. Nevertheless, important comorbidities such as osteoarthritis, several cancers (e.g., colorectal, endometrial), and mental health disorders were not included, potentially leading to underestimation of the full clinical and economic benefits of treatment.

Several limitations should be acknowledged. First, although semaglutide's efficacy estimates were derived from the robust STEP 8 trial, long-term extrapolation of weight trajectories and complication risks required assumptions and the application of published risk equations. These may not fully reflect real-world outcomes in Greece and, given the BMI-only surrogate approach, may underestimate pathways not directly mediated through BMI. Second, tirzepatide, a dual GIP/GLP-1 receptor agonist with superior weight-loss efficacy, was not considered. Although it is currently available in Greece only on an out-of-pocket basis, its potential future reimbursement makes it a key comparator for subsequent analyses. Third, this analysis was conducted from the perspective of the Greek third-party payer (EOPYY) and therefore excluded indirect costs, such as productivity losses, absenteeism, presenteeism, and premature mortality, associated with obesity. As these costs represent a substantial component of the overall economic burden of obesity, excluding a societal perspective may underestimate the broader economic benefits of effective pharmacotherapy. Fourth, although STEP-8 provides the only head-to-head randomized evidence directly comparing semaglutide and liraglutide, its trial population may not fully reflect the demographic and clinical profile of Greek patients with obesity. Given the limited availability of comprehensive local data, some differences between the modeled cohort and the real-world Greek population may remain unaccounted for, which should be considered when interpreting the findings. Fifth, while the STEP-8 trial primarily informed baseline characteristics, specific parameters (such as smoking prevalence, hypertension, and diabetes distribution) were supplemented with expert clinical input to reflect the Greek population more accurately. Although this approach enhances local relevance, it may introduce a degree of subjectivity and should be interpreted as a potential source of uncertainty. Finally, although this analysis incorporated more comorbidities than many prior studies, several obesity-related conditions (e.g., certain cancers, osteoarthritis, and mental health disorders) were not included. Similarly, bariatric surgery was not modeled as a comparator or as an acute event manifesting across health states. These omissions suggest that our results may be conservative estimates of semaglutide's cost-effectiveness profile.

Future research should expand on these findings in several directions. First, the application of alternative frameworks, such as the Core Obesity Model (20–23), would allow for the validation of results across different structural assumptions and the incorporation of additional risk factors beyond BMI, thereby enabling more accurate long-term projections. Second, societal-perspective analyses in Greece are warranted to capture the broader economic implications of obesity management, including productivity losses and informal care costs. Third, head-to-head evaluations of semaglutide and emerging therapies, particularly tirzepatide, will be crucial in informing future reimbursement and treatment decisions. Fourth, the integration of real-world data on adherence, weight trajectories, safety, and long-term outcomes in Greek patients will strengthen the external validity of future models. Finally, subgroup analyses in patients with obesity and coexisting conditions such as type 2 diabetes or non-alcoholic steatohepatitis could provide deeper insights into the dual benefits of obesity management for both metabolic and hepatic outcomes.

In summary, this study provides robust evidence that semaglutide 2.4 mg is a cost-effective treatment for obesity in Greece compared with liraglutide 3.0 mg. These results have important implications for reimbursement and resource allocation in Greece, where obesity imposes a substantial health and economic burden, and support the expansion of access to clinical and cost-effective pharmacotherapies such as semaglutide.

## 5 Conclusion

The present cost-effectiveness analysis demonstrates that semaglutide 2.4 mg is a cost-effective option compared with liraglutide 3.0 mg for adults with obesity (BMI ≥ 35 kg/m^2^ and at least one weight-related comorbidity) in Greece, at a willingness-to-pay threshold of €27,117 per QALY gained. Deterministic and probabilistic sensitivity analyses confirmed the robustness of these findings.

## Data Availability

The original contributions presented in the study are included in the article/Supplementary material, further inquiries can be directed to the corresponding author.
