# Supplementary material for: Cost-effectiveness of semaglutide 2.4 mg versus liraglutide 3 mg for the treatment of obesity in Greece
Source: Front Public Health. 2025 Oct 28;13:1690211. doi: 10.3389/fpubh.2025.1690211 (PMC12602536; doi:10.3389/fpubh.2025.1690211)
Supplement: Supplementary file 1 [file Supplementary_file_1.docx]

Supplementary Material

**Table 1: Key clinical efficacy and safety inputs**

|  | Semaglutide 2.4 mg | | Liraglutide 3 mg | |
| --- | --- | --- | --- | --- |
|  | Mean | SE | Mean | SE |
| Percent (%) weight loss vs. baseline in cycles 2 and 3 [months 4-9] | -11.4% | 0.29% | -9.3% | 0.26% |
| Percent (%) weight loss vs. baseline in cycle 4 [months 10-12] | -19.0% | 0.49% | -8.8% | 0.25% |
| Percent (%) weight loss vs. baseline in cycle 5 [months 13-24] | -19.0% | 0.49% | -8.8% | 0.25% |
| Percent (%) weight loss vs. baseline in cycle 6 [months 25-36] | -19.0% | 0.49% | -8.8% | 0.25% |
| Proportion not achieving early responder status defined as >=5% weight loss from baseline | 24.60% | 7.7% | 38.58% | 7.0% |
| Catch-up time after treatment discontinuation | 1 | 0 | 1 | 0 |
| Percent (%) weight loss at 1 year - average bariatric surgery | -27.7% | 0.07% | -27.7% | 0.07% |
| Natural weight increases per year (kg) | 0.48 | 0.12 | 0.48 | 0.12 |
| Maximum age until weight increases (yrs) | 75.00 | 17.00 | 75.00 | 17.00 |
| Non-severe hypoglycaemia | 0.02% | - | 0.8% | - |
| Severe hypoglycaemia | 7.9% | - | 11% | - |
| Severe gastrointestinal | 0.067% | - | 0.026% | - |

Table 2: Hazard Ratios for mortality by BMI level.

| BMI (Kg/m^2^) | HRs | BMI (Kg/m^2^) | HRs | BMI (Kg/m^2^) | HRs | BMI (Kg/m^2^) | HRs |
| --- | --- | --- | --- | --- | --- | --- | --- |
| 15 | 2.25 | 25 | 0.86 | 35 | 1.34 | 45 | 2.42 |
| 16 | 1.99 | 26 | 0.84 | 36 | 1.44 | 46 | 2.51 |
| 17 | 1.76 | 27 | 0.84 | 37 | 1.54 | 47 | 2.60 |
| 18 | 1.56 | 28 | 0.86 | 38 | 1.65 | 48 | 2.67 |
| 19 | 1.39 | 29 | 0.90 | 39 | 1.77 | 49 | 2.74 |
| 20 | 1.24 | 30 | 0.94 | 40 | 1.88 | 50 | 2.79 |
| 21 | 1.12 | 31 | 1.00 | 41 | 1.99 | 51 | 2.83 |
| 22 | 1.02 | 32 | 1.07 | 42 | 2.11 | 52 | 2.83 |
| 23 | 0.95 | 33 | 1.15 | 43 | 2.21 |  |  |
| 24 | 0.89 | 34 | 1.24 | 44 | 2.32 |  |  |

BMI – body mass index; HR – Hazard ratio

**Table 3: Coefficients applied in the model for the baseline utility calculation**

| Variable | β coefficient | 95% CI Lower | 95% CI Upper |
| --- | --- | --- | --- |
| Intercept | 0.942975 | 0.573 | 1.313 |
| Age (years) | -0.0005414 | -0.000754 | -0.000329 |
| Heart/circulatory disease (excl. HTN) | -0.0742818 | -0.103 | -0.045 |
| Hypertension | -0.0097531 | -0.0136 | -0.0059 |
| Smoking – Current | 0.0039044 | 0.00237 | 0.00544 |
| Smoking – Previous | -0.0081972 | -0.0114 | -0.0050 |
| Smoking – Never | 0 | 0 | 0 |
| BMI (linear term) | 0.0065954 | 0.0040 | 0.0092 |
| BMI² (quadratic term) | -0.0002476 | -0.00034 | -0.00015 |
| BMI³ (cubic term) | 0.00000175 | 0.0000011 | 0.0000024 |
| Prediabetes | -0.0031133 | -0.0043 | -0.0020 |

**Table 4: Cost decomposition in the base case and probabilistic sensitivity analysis**

| Cost category | Deterministic incremental (€) | PSA mean incremental (€) |
| --- | --- | --- |
| Obesity drug acquisition | 1,417.42 | 997.23 |
| Obesity monitoring | 6.05 | 4,25 |
| Obesity complications — state costs | -337.31 | -1,287.99 |
| Obesity complications — event costs | −2.79 | -14.14 |
| Total incremental cost | 1,083.37 | −300.65 |
